# Cucurbitacin E Has Neuroprotective Properties and Autophagic Modulating Activities on Dopaminergic Neurons

**DOI:** 10.1155/2014/425496

**Published:** 2014-12-09

**Authors:** Anne-Marie Arel-Dubeau, Fanny Longpré, Julie Bournival, Cindy Tremblay, Julie Demers-Lamarche, Pavlina Haskova, Everaldo Attard, Marc Germain, Maria-Grazia Martinoli

**Affiliations:** ^1^Cellular Neurobiology, Department of Medical Biology, Université du Québec à Trois-Rivières, Trois-Rivières, QC, Canada G9A 5H7; ^2^Department of Biochemical Sciences, Faculty of Pharmacy, Charles University in Prague, 500 50 Hradec Kralove, Czech Republic; ^3^Institute of Earth Systems, University of Malta, Msida MSD 2080, Malta; ^4^Department of Psychiatry and Neuroscience, Laval University and CHU Research Center, Québec, QC, Canada G1W 1C2

## Abstract

Natural molecules are under intensive study for their potential as preventive and/or adjuvant therapies for neurodegenerative disorders such as Parkinson's disease (PD). We evaluated the neuroprotective potential of cucurbitacin E (CuE), a tetracyclic triterpenoid phytosterol extracted from the *Ecballium elaterium* (Cucurbitaceae), using a known cellular model of PD, NGF-differentiated PC12. In our postmitotic experimental paradigm, neuronal cells were treated with the parkinsonian toxin 1-methyl-4-phenylpyridinium (MPP^+^) to provoke significant cellular damage and apoptosis or with the potent *N,N*-diethyldithiocarbamate (DDC) to induce superoxide (O_2_^•−^) production, and CuE was administered prior to and during the neurotoxic treatment. We measured cellular death and reactive oxygen species to evaluate the antioxidant and antiapoptotic properties of CuE. In addition, we analyzed cellular macroautophagy, a bulk degradation process involving the lysosomal pathway. CuE showed neuroprotective effects on MPP^+^-induced cell death. However, CuE failed to rescue neuronal cells from oxidative stress induced by MPP^+^ or DDC. Microscopy and western blot data show an intriguing involvement of CuE in maintaining lysosomal distribution and decreasing autophagy flux. Altogether, these data indicate that CuE decreases neuronal death and autophagic flux in a postmitotic cellular model of PD.

## 1. Introduction

Mitochondrial dysfunction has been recently recognized to contribute to the onset of many neurodegenerative diseases characterized by protein aggregation, cellular inclusions, impairment of metabolic functions, and cellular trafficking [1–3]. In Parkinson's disease (PD), for example, these effects lead to the degeneration of the nigrostriatal dopaminergic (DAergic) pathway characterized by the selective death of DAergic neurons and dopamine content depletion [4]. The hypotheses regarding the etiology of PD pinpoint mitochondrial defects and oxidative stress, ranging from mutations in proteins regulating mitochondrial turnover to functional impairment of the respiratory chain, as major causes of neurodegeneration [5, 6]. Moreover, formation of the characteristic protein inclusions of PD, Lewy bodies, illustrates the challenge of protein misfolding and aggregation, not rescued by cellular degradation mechanisms [7].

The autophagy-lysosomal degradation pathway, which is essential for turnover of mitochondria (mitophagy) and degradation of aggregated proteins (aggrephagy), has been linked with the development of neurodegenerative disorders [8–11]. Its impairment leads to formation of protein inclusions and accumulation of damaged mitochondria, a major source of reactive oxygen species (ROS) [12, 13]. ROS can then oxidize proteins, eventually inducing their misfolding and aggregation, and trigger the apoptotic cascade. Protein inclusions and accumulation of damaged mitochondria have been observed for decades in the brains of PD patients [4, 14]. Impaired autophagy has also been observed in animal models of PD and in human postmortem brain sections [15, 16]. Mutations in genes associated with familial PD, such as parkin, PINK1, LRRK2, and DJ-1, impair mitochondrial turnover and protein aggregate clearance [17–20]. Hence, the accumulation of protein aggregates and toxic mitochondria in the cytoplasm may prompt increased oxidative stress and, over time, cell death.

Impairment of the respiratory chain complex I found in PD brains [21, 22] is reproduced by treatment with 1-methyl-4-phenylpyridinium (MPP^+^), the active metabolite of 1-methyl-4-phenyl-1,2,3,6-tetrahydropyridine (MPTP), a neurotoxin which causes a Parkinson-like syndrome in many model organisms and in humans [23–25]. MPP^+^ crosses the blood-brain barrier and is converted into toxic MPP^+^ in astrocytes by the enzyme monoamine oxidase B [4]. It is then incorporated by dopaminergic (DAergic) neurons via the dopamine transporter (DAT) and interferes directly with complex I of the respiratory chain in the mitochondrion [26, 27], leading to production of ROS, oxidative stress, and apoptosis.

In the last decade, a growing number of studies have revealed several natural molecules possessing interesting antioxidant and antiapoptotic properties in neuronal cell culture and in animal models of neurodegeneration that may improve cognitive health in humans [23, 28–35]. Among them, cucurbitacins, triterpene steroids extracted mainly from the Cucurbitaceae plant family, have been studied for their antitumoral, anti-inflammatory, and antioxidant properties [36–40]. In particular, cucurbitacin E (CuE) has been reported to possess anti-inflammatory and antiproliferative properties [41], effects mediated by its action on the polymerisation of the actin cytoskeleton [42–45].

The aim of this work was to evaluate CuE as a preventive treatment for the onset of neuronal death induced by the potent parkinsonian toxin MPP^+^ in a neuronal DAergic model. We first assessed CuE against MPP^+^-induced cellular death. Then, we examined the antioxidant properties of CuE, and finally we investigated the autophagic degradation pathway as a potential mechanism for CuE-mediated neuroprotection. The modulation of autophagy by CuE suggests that this could contribute to its neuroprotective effect of CuE. To our knowledge this is the first time that CuE is used in a postmitotic neuronal model and shows neuroprotective and autophagy regulating properties.

## 2. Materials and Methods

### 2.1. Drugs and Chemicals

All reagents and chemicals were purchased from Sigma (St. Louis, MO) unless stated otherwise.

### 2.2. Cucurbitacin E Extraction and Purification

CuE was extracted from the fruit juice of* Ecballium elaterium* (L.) A.Rich., collected from Marsascala (Malta) with a yield of 52% from the prepared elaterin. Briefly, the fruit juice was dried at 40°C and extracted with CHCl_3_ (5 mL) and then mixed with an equal volume of petroleum ether. The filtrate was dissolved in absolute EtOH and then filtered through a 0.22 *μ*m pore size membrane. The purity of the compound (98.24%) was analysed by spectroscopy and HPLC [46].

### 2.3. Cell Culture and Treatments

**Figure 1:**
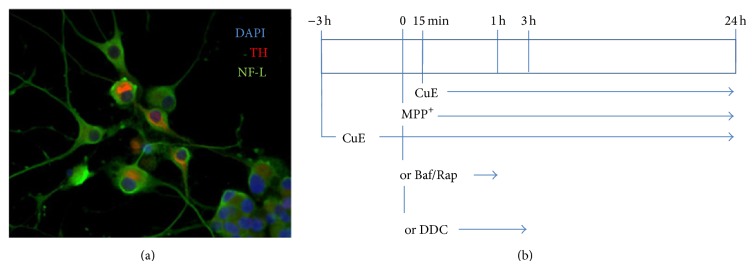
(a) Immunofluorescence picture revealing the neuronal phenotype of PC12 cells after 5 days of NGF treatment. TH: tyrosine hydroxylase, as marker of dopamine; NFL: neurofilament-low-chain, as a marker of neuronal cytoskeleton. Nuclei were counterstained with DAPI. (b) Experimental set-up. CuE 10^−10^ M was administered 3 hours before MPP^+^ or DDC or bafilomycin (Baf) or rapamycin (Rap) treatment. CuE was administered once more 15 min after MPP^+^ or DDC or Baf or Rap.

**Figure 2:**
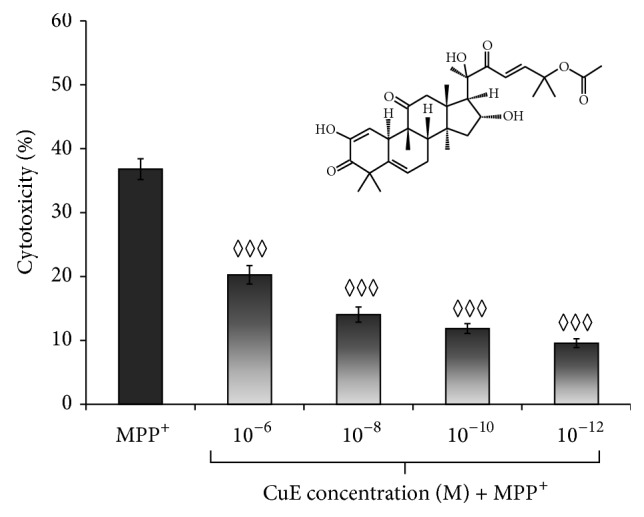
Dose-response studies. Cells were pretreated with CuE concentrations ranging from 10^−6^ to 10^−10^ M for 3 h and then exposed to 5 mM MPP^+^. Cytotoxicity was measured using the colorimetric LDH release assay. The absorbance value obtained for the untreated control was subtracted from all other values, as described in Materials and Methods. Administration of CuE alone induced minimal cellular death. Values are the average of 6 samples from 4 independent experiments for a total of 24 measurements. Data are expressed as means ± S.E.M. *n* = 4. ^◊◊◊^ *P* < 0.001 versus MPP^+^.

**Figure 3:**
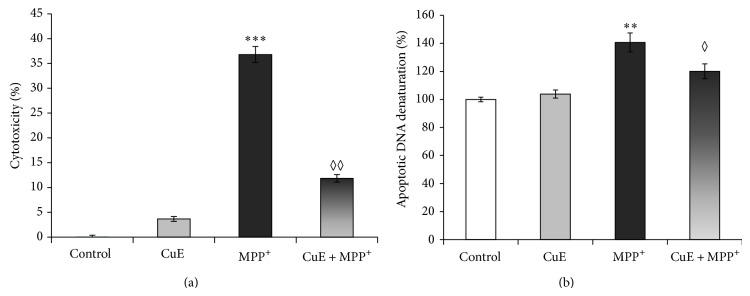
(a) Effects of CuE on cytotoxicity induced by MPP^+^ in neuronal PC12 cells, as measured by colorimetric LDH release assay. The absorbance value obtained for the untreated control was subtracted from all other values, as described in Materials and Methods. MPP^+^ treatment shows an important enhancement of cellular death, compared to control condition. Administration of CuE powerfully rescues MPP^+^-induced cellular death. Values are the average of 6 samples from 4 independent experiments for a total of 24 measurements. Data are expressed as means ± S.E.M. *n* = 4. ^***^ *P* < 0.001 versus Ctrl and ^◊◊^ *P* < 0.01 versus MPP^+^. (b) Detection of DNA denaturation using monoclonal antibodies against single-stranded DNA. Neuronal PC12 cells treated with MPP^+^ alone show a significant increase in cell death compared to control (Ctrl). Administration of CuE prior to MPP^+^ (CuE + MPP^+^) shows a decrease in DNA denaturation compared to MPP^+^ alone. Values are the average of 6 samples from 3 independent experiments for a total of 18 measurements. Data are expressed as means ± S.E.M. *n* = 4. ^**^ *P* < 0.01 versus Ctrl and ^◊^ *P* < 0.05 versus MPP^+^.

PC12 cells, obtained from the American Type Culture Collection (Rockville, MD), were maintained in a humidified environment at 37°C and 5% CO_2_ atmosphere. They were grown in Dulbecco's modified Eagle's medium (DMEM) supplemented with 10% (v/v) heat-inactivated horse serum (HS), 5% (v/v) heat-inactivated fetal bovine serum (FBS), and gentamicin (50 *μ*g/mL). Neuronal PC12 cell differentiation was evoked by nerve growth factor-7S (NGF, 50 ng/mL) in DMEM supplemented with 1% FBS for 5 days (Figure 1(a)), as already described in [28, 47]. In these conditions, 85% of naïve PC12 differentiated into neuronal cells. Treatments with CuE and MPP^+^ were performed in DMEM medium without phenol red supplemented with 1% charcoal-stripped FBS. Cells were pretreated with CuE 10^−10 ^M for 3 h and then exposed to 5 mM or 500 *μ*M MPP^+^ for 24 h [48]. CuE 10^−10 ^M was added once more for 15 min after MPP^+^ administration, as illustrated in Figure 1(b). Neuroprotective CuE concentration was determined by dose-response experiments (Figure 2). Administration of CuE alone induces only minimal cellular death (Figure 3). Bafilomycin A_1_ (Baf) 100 nM, inhibiting lysosomal acidification, was used to assess autophagic flux [49, 50]. Rapamycin (Rap) (500 nM), an inhibitor of mammalian target of rapamycin (mTOR), was used as an inducer of autophagy [51]. Both were used separately on live cells 1 hour prior to extraction or fixation (Figure 1(b)). All experiments were performed in charcoal-stripped serum to remove steroids from the medium.

### 2.4. Cytotoxicity Measurements

Cytotoxicity was evaluated by a colorimetric assay based on the measurement of supernatant lactate dehydrogenase (LDH) activity, as already described in [28]. The amount of enzyme activity detected in culture supernatant correlates with the portion of lysed cells [52]. Briefly, 50 *μ*L of cell-free supernatant was harvested to quantify LDH activity by measuring absorbance at a wavelength of 490 nm with a microplate reader (Thermolab Systems, Franklin, MA). Total cellular LDH was determined by lysing the cells with 1% Triton X-100 (high control); the assay medium served as the low control and was subtracted from all absorbance measurements:

(1) $$\begin{array}{c} \text{Cytotoxicity}\;\left(\%\right)=\frac{\text{experimental}\;\;\text{value}-\text{low}\;\;\text{control}}{\text{high}\;\;\text{control}-\text{low}\;\;\text{control}}\times 100. \end{array}$$

### 2.5. Specific DNA Denaturation Detection

Specific detection of DNA denaturation was studied using the single-stranded DNA (ssDNA) apoptosis ELISA kit (Chemicon International, Billerica, MA), as already reported in [47, 53]. This procedure is based on selective DNA denaturation by formamide and heat in apoptotic cells that does not occur in necrotic cells or in cells with DNA breaks in the absence of apoptosis [54]. The detection of denatured DNA was performed with a monoclonal antibody highly specific to ssDNA and a peroxidase-labelled secondary antibody on fixed neuronal PC12 cells, seeded at 25 000 cells/cm^2^ in 96-well plates. The reaction was then stopped with a hydrochloric acid solution and ssDNA was quantified by measuring absorbance at 405 nm in a microplate reader (Thermolab Systems). ssDNA was analyzed with reference to control conditions. Absorbance of positive (wells coated with ssDNA) and negative controls (wells coated with S1 nuclease) served as quality controls for the ELISA assay, as previously described in [47, 53].

### 2.6. ROS Detection

The antioxidant effect of CuE against MPP^+^-induced ROS production was evaluated by the dihydrorhodamine 123 (DHR) assay and MitoSOX Red (Invitrogen, Burlington, ON, Canada), according to a previously described method [55]. Briefly, to detect OH^•^, NO_2_^•^, CO_3_^•−^, H_2_O_2_, HOCl, and ONOO^−^ by DHR [56, 57], NGF-differentiated PC12 cells were grown and treated on collagen-coated circular glass coverslips. Then, they were quickly washed with PBS 0.1 M and exposed to 250 *μ*L of DHR at 37°C for 20 min. Slides with live cells were immediately examined under a Leitz Orthoplan fluorescence microscope (Leica, Wetzlar, Germany) and photographed with a QImaging camera (Nikon, Mississauga, ON, Canada), as already described in [55]. Fluorescence intensity was measured using NIS Elements 2.2 software (Nikon).

The antioxidant effects of CuE against mitochondrial superoxide anion (O_2_^•−^) production were evaluated with MitoSOX Red, as already described in [47, 55]. NGF-differentiated PC12 cells were washed with Hank's buffered salt solution (HBSS) and incubated for 10 min at 37°C with a 5 *μ*M solution of MitoSOX Red. Nuclei were counterstained with Hoechst 33342 (5 *μ*g/mL) for 15 min at 37°C, and then cells were fixed for 20 min in 4% paraformaldehyde at 37°C, mounted on glass slides with ProLong Antifade kit (Invitrogen), examined under a Leitz Orthoplan fluorescence microscope (Leica), and photographed with a QImaging camera (Nikon). Fluorescence intensity was measured using NIS Elements 2.2 software (Nikon). To demonstrate MitoSOX Red selectivity, a positive control was performed using sodium diethyldithiocarbamate (DDC), a superoxide dismutase (SOD) inhibitor, in control medium.

### 2.7. Immunofluorescence

NGF-differentiated PC12 cells were grown and treated on collagen-coated circular glass coverslips in 24-well plates. Cells were fixed for 20 min at 37°C in 4% paraformaldehyde, then washed, and incubated for 1 h at room temperature (RT) in a blocking and permeabilizing solution, as already described in [47, 55].

For lysosomes and mitochondria detection, coverslips were incubated overnight with lysosome-associated membrane protein-2a (LAMP2, Novus Biologicals NBP1-95696) and with mitochondria-specific heat-shock protein-70 (mtHSP70, ABR Bioreagents MA3-28) antibodies, respectively, followed by fluorescent secondary antibodies (Alexa Fluor 488 and 594, Jackson ImmunoResearch). Slides were examined under a Zeiss confocal microscope using ZEN Imaging software (Zeiss, Toronto, ON, Canada).

Immunofluorescence detection of the cellular cytoskeleton was performed by exposing fixed cells with anti-actin 1 : 1,000 (Sigma A2066), anti-*β*3-tubulin 1 : 1,000 (Santa Cruz Biotechnology sc-80005), and anti-neurofilament-M 1 : 3,000 (NF-M, Sigma N5264) antibodies followed by the appropriate anti-rabbit fluorescein isothiocyanate (FITC, Sigma F0382) or anti-mouse Cy3 (Sigma C2181) fluorescent secondary antibodies and DAPI nuclear staining. Slides were examined under a Zeiss confocal microscope using ZEN Imaging software (Zeiss).

Recently, detecting microtubule-associated protein 1A/1B-light chain 3 (LC3) has become a reliable method for monitoring autophagy and autophagy-related processes [58]. The amount of LC3-II is clearly correlated with the number of autophagosomes [59]. We used immunofluorescence on cells grown and treated on coverslips. Cells were then exposed overnight with a primary antibody for LC3b 1 : 250 (Cell Signaling Techn. 3868) at 4°C. After washing with PBS, cells were exposed to the fluorophore-conjugated secondary antibody, anti-mouse Cy3 (Invitrogen A-21467). Finally, coverslips were mounted on glass slides with ProLong Antifade kit (Invitrogen) and examined under an Olympus Corporation FV1200S confocal microscope using Fluoview 10-ASW 4.0 software (Olympus, Richmond Hill, ON, Canada). Percentage of cell occupied by puncta stained with LC3b (area fraction) was quantified using ImageJ free software (http://imagej.nih.gov/).

### 2.8. Analysis of Autophagosomes

The vital dye acridine orange and the specific autophagy epifluorescent dye Cyto-ID (Enzo Life Sciences) were used for autophagy detection. Acridine orange is a lysotropic dye that accumulates in late acidic autophagic vacuoles [60]. Cyto-ID is dye for autophagosomes [61–63].

NGF-differentiated PC12 cells were seeded at 25 000 cells/cm^2^ and acridine orange or Cyto-ID staining was performed immediately after experimental treatments on live cells, according to the manufacturer's instructions. Positive control for autophagy was rapamycin (500 nM), a known mTOR inhibitor and autophagy inducer. All coverslips were rinsed with PBS and nuclei counterstained with Hoechst 33342 (5 *μ*g/mL); then cells were fixed with 4% paraformaldehyde and mounted on glass slides with ProLong Antifade kit (Invitrogen). Cells were observed with a Leitz Orthoplan microscope (Leica) and photographed with a QImaging camera (Nikon). Fluorescence intensity was measured by NIS Elements 2.2 software (Nikon) for acridine orange. Vacuoles larger than 1 *μ*m stained with Cyto-ID were blindly counted on 10 different optic fields from at least 3 slides per group [48] using ImageJ free software (http://imagej.nih.gov/).

### 2.9. Electrophoresis and Immunoblot Analysis

NGF-differentiated PC12 cells were grown and treated in collagen-coated 6-well plates. Total cellular proteins were extracted using nuclear extraction kit (Active Motif, Carlsbad, CA, USA). Cells were lysed and protein concentration was quantified by BCA protein assay kit (Pierce, Rockford, IL, USA). Equal amounts of proteins were loaded onto 10% sodium dodecyl sulphate polyacrylamide gels. After electrophoretic separation (125 V, for 1 h30), proteins were transferred onto PVDF membranes (0.22 *μ*m pore size, BioRad, Mississauga, ON, Canada) at 25 V overnight. The membranes were blocked for 30 min to 1 h at RT and incubated overnight at 4°C with primary antibodies, anti-p62, a cargo protein, 1 : 1000 (Progen Biotechnik GmbH, GP62-C), and antityrosine hydroxylase (TH) 1 : 2000. Membranes were then incubated with horseradish peroxidase-conjugated secondary antibodies 1 : 10,000 for 1 h30 at RT. Immunopositive signals were visualized by enhanced chemiluminescence with the AlphaEaseFC imaging system (Alpha Innotech, San Leandro, CA, USA) and analyzed with AlphaEaseFC software (Alpha Innotech) and ImageJ (http://imagej.nih.gov/). TH is the enzyme responsible for the conversion of L-tyrosine to L-3,4-dihydroxyphenylalanine (L-DOPA) which is the precursor for dopamine. TH is found ubiquitously in neuronal differentiated PC12 and was used to normalise the p62 signal as in our experiments CuE does not modulate TH expressions (data not shown).

### 2.10. Statistical Analysis

Significant differences between groups were ascertained by one-way analysis of variance (ANOVA), followed by Tukey's post hoc analysis, achieved with the GraphPad InStat program, version 3.06 for Windows (http://www.graphpad.com/). All data, analyzed at the 95% confidence interval, were expressed as means ± S.E.M. from at least 3 independent experiments. Asterisks indicate statistical differences between MPP^+^ and control (^***^ *P* < 0.001, ^**^ *P* < 0.01, and ^*^ *P* < 0.05) and diamonds denote statistical differences between the treatment and MPP^+^ or Baf condition (^◊◊◊^ *P* < 0.001, ^◊◊^ *P* < 0.01, and ^◊^ *P* < 0.05).

## 3. Results

### 3.1. CuE Treatment Reduces MPP^+^-Induced Cell Death

The neuroprotective effects of CuE in PC12 neuronal cells (Figure 1(a)) treated with MPP^+^ were assessed by measuring LDH release. Experimental CuE concentrations were determined by a dose-response assay aimed at finding the lowest neuroprotective dose of CuE in neuronal PC12 cells (Figure 2). After a 24 h treatment with 5 mM MPP^+^, cell death was significantly higher in MPP^+^-treated cells than in cells treated with MPP^+^ and CuE concentrations ranging from 1 *μ*M to 1 pM (Figure 2).  Figure 3(a) shows that treatment with 10^−10^ M CuE reduced MPP^+^-induced cellular death (CuE + MPP^+^) by 70%, while treatment with CuE alone minimally affected cell death (CuE).

Neuronal death was also detected by a specific DNA denaturation assay (Figure 3(b)). After a 24 h treatment with 500 *μ*M MPP^+^, specific apoptotic DNA denaturation increases by 44% compared to control condition (Ctrl). CuE administration reduced significantly the amount of DNA denaturation (CuE + MPP^+^) while CuE treatment alone caused no significant changes (Figure 3(b)).

### 3.2. CuE Does Not Significantly Lower Oxidative Stress

**Figure 4:**
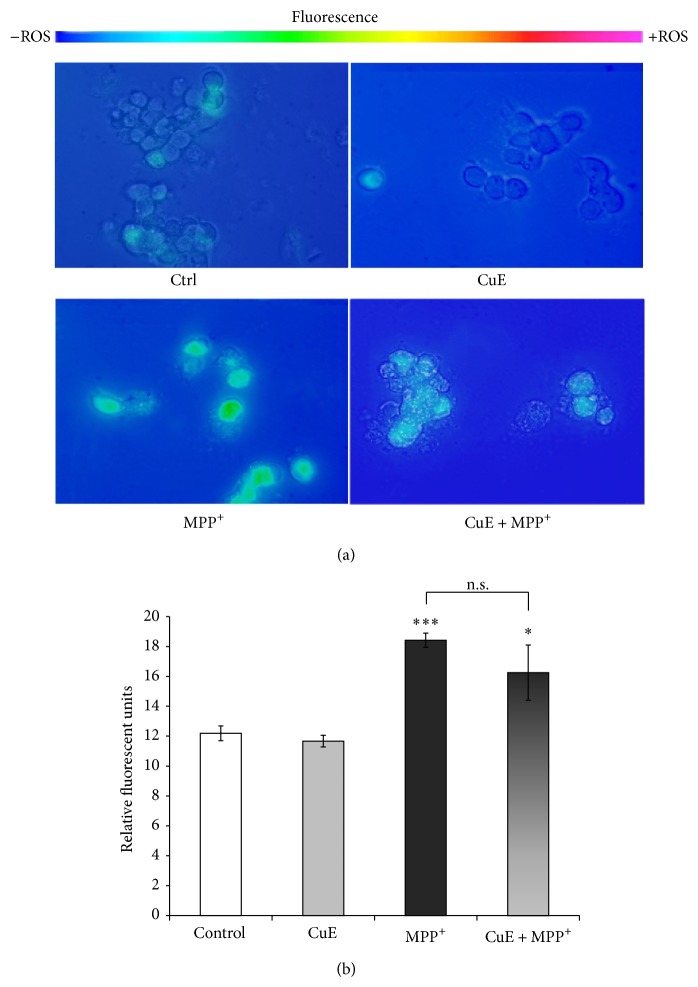
Rhodamine detection of ROS in neuronal PC12 cells after MPP^+^ and/or CuE treatment. Nonfluorescent DHR is converted to fluorescent rhodamine in the presence of several free radicals (OH^•^, NO_2_^•^, CO_3_^•−^, H_2_O_2_, HOCl, and ONOO^−^). (a) Fluorescent microscopy. A significant signal is marked in neuronal cells treated with MPP^+^ but not in those exposed to CuE or the vehicle (Ctrl). Administration of CuE + MPP^+^ does not reduce fluorescence significantly compared to MPP^+^ alone. Scale bar = 10 *μ*m. (b) Histogram. Semiquantitative analysis of rhodamine fluorescence. Data are expressed as relative fluorescence units and are means ± S.E.M. *n* = 4. ^***^ *P* < 0.001 versus Ctrl and n.s. = nonsignificant.

DHR is oxidized to fluorescent rhodamine by free radicals such as OH^•^, NO_2_^•^, CO_3_^•−^, H_2_O_2_, HOCl, and ONOO^−^ [56, 57]. Figures 4(a) and 4(b) illustrate that fluorescence levels were significantly increased after MPP^+^ treatment compared to control conditions, supporting the oxidative nature of the MPP^+^ neurotoxin [47, 55]. CuE alone did not modulate rhodamine fluorescence and the administration of CuE with MPP^+^ (CuE + MPP^+^) failed to significantly lower free radical levels compared to MPP^+^ alone, in our postmitotic neuronal paradigm (Figures 4(a) and 4(b)).

**Figure 5:**
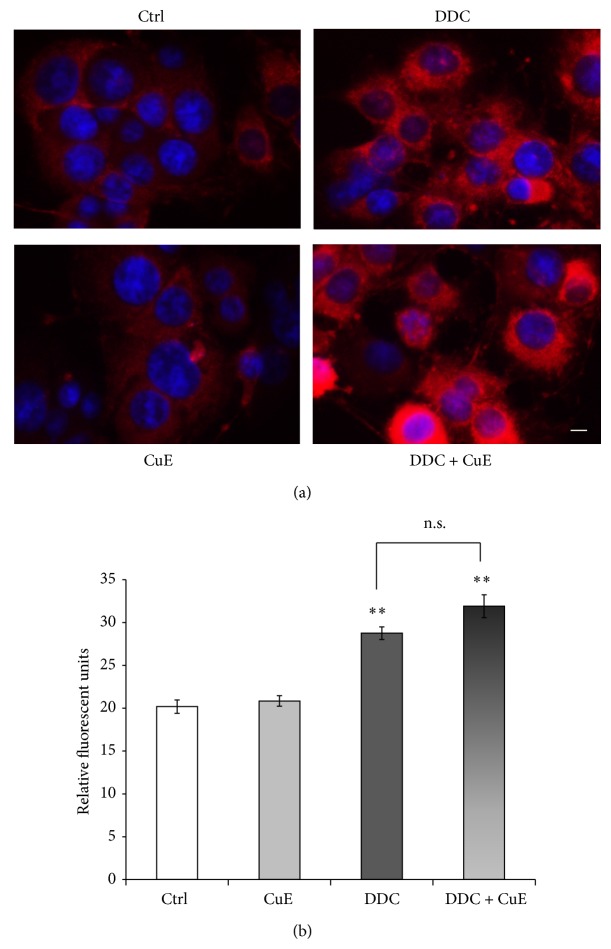
Selective detection of superoxide anion by MitoSOX Red. This fluorogenic dye enters the cell and is oxidized by O_2_^•−^ to a red fluorescent molecule. DDC, a specific superoxide dismutase inhibitor, was used as a positive control for O_2_^•−^ production. (a) Fluorescence microphotographs show intense MitoSOX Red signal in DDC exposed cells. CuE does not rescue cells from oxidative stress as O_2_^−^ levels are equally high in DDC condition with or without CuE pretreatment. Untreated control and CuE-only condition show similar low fluorescence levels. Nuclei were counterstained in blue with Hoechst 33342. Scale bar = 10 *μ*m. (b) Histogram. Semiquantitative measures of MitoSOX Red fluorescence. Data are expressed as relative fluorescence units and are means ± S.E.M. *n* = 3. ^**^ *P* < 0.01 versus Ctrl. n.s. = nonsignificant.

To further study the antioxidant potential of CuE, we used MitoSOX Red to measure its ability to quench ROS generated by the inhibition of superoxide dismutase (SOD) (Figures 5(a) and 5(b)). Cells were treated with or without vehicle, CuE or DDC, a potent SOD inhibitor, for 3 h, as previously reported in [47, 55]. This time period was chosen since free radical generation and oxidative stress are early events in the causative process of cellular death [64, 65] and experimentally generated the best detectable superoxide levels [47, 55]. Figures 5(a) and 5(b) show that fluorescence levels were, as expected, the highest in DDC-treated cells and that CuE treatment could not revert the superoxide anion levels induced by the inhibition of SOD (DDC + CuE), thus suggesting that CuE had no preventive effect on superoxide anion levels. Control conditions display low levels of red fluorescence and CuE treatment alone shows similar low levels.

### 3.3. CuE Regulates the Presence of Autophagic Vacuoles

**Figure 6:**
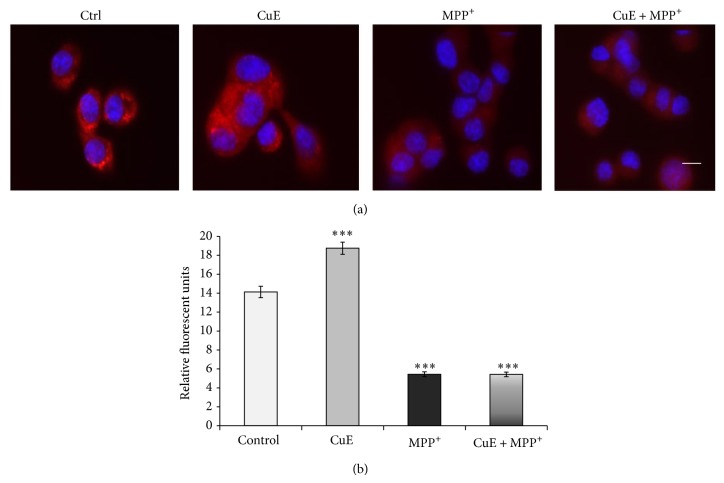
Acridine orange detection of acidic lysosomes. Acridine orange enters acidic compartments such as lysosomes and becomes protonated and sequestered. (a) Microphotographs illustrating an intense fluorescence when CuE is administered alone and low of fluorescence in MPP^+^ or CuE + MPP^+^ conditions. Nuclei were counterstained in blue with Hoechst 33342. Scale bar = 10 *μ*m. (b) Histogram. Semiquantitative measures of acridine orange fluorescence. Data are expressed as relative fluorescence units and are means ± S.E.M. *n* = 3. ^***^ *P* < 0.001 versus Ctrl.

**Figure 7:**
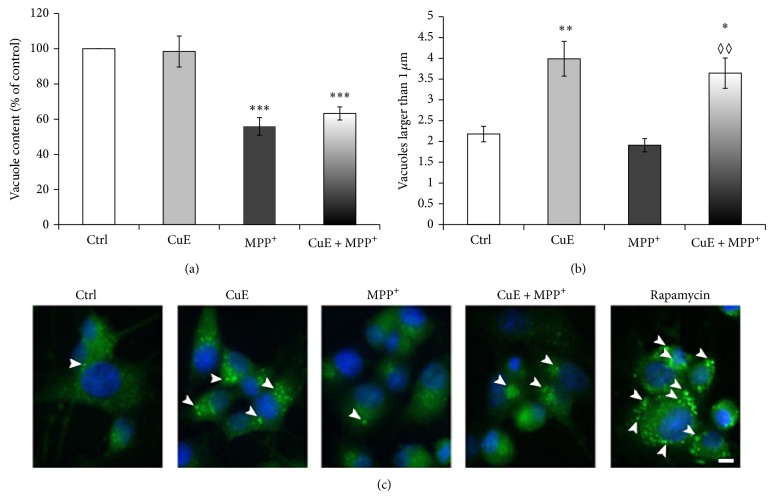
Epifluorescence analysis of autophagy in neuronal PC12 cells treated with CuE and/or MPP^+^ using Cyto-ID autophagy detection kit. (a) Histogram. Total content of vacuoles positive for Cyto-ID. CuE administration could not change the total number of vacuoles while MPP^+^ considerably decreases cellular vacuole content. (b) Histogram. Number of vacuoles larger than 1 *μ*m per cell. Administration of CuE alone clearly induces an increased number of larger than 1 *μ*m vacuoles. Administration of MPP^+^ intensely reduces the presence of these large vacuoles. Pretreatment with CuE before MPP^+^ (CuE + MPP^+^) still shows similar levels of large vacuoles as in CuE-alone treatment. (c) Fluorescent microscopy. CuE-treated cells show an increased number of autophagic vacuoles larger than 1 *μ*m (arrowheads). Positive control for autophagy was rapamycin, a known autophagy inducer. Nuclei were counterstained in blue with Hoechst 33342. Scale bar = 10 *μ*m. Data are expressed as number of vacuoles larger than 1 *μ*m per cell and are means ± S.E.M. *n* = 3. ^**^ *P* < 0.01 and ^*^ *P* < 0.05 versus Ctrl. ^◊◊^ *P* < 0.01 versus MPP^+^ and n.s. = nonsignificant.

Since CuE acts as a neuroprotective molecule but does not play an antioxidant role to promote neuronal survival in our postmitotic model, we examined whether CuE could modulate autophagy, a cellular process allowing the degradation and recycling of cellular components, thus playing an important role in neuronal survival. We first analysed the effect of CuE and MPP^+^ on the presence of acidic vesicles as illustrated by acridine orange red fluorescence (Figures 6(a) and 6(b)). Consistent with a depletion of lysosomes by MPP^+^ [66], Figures 6(a) and 6(b) illustrate a dramatic decrease in acridine orange-positive vesicles when PC12 neuronal cells were exposed to MPP^+^. In contrast, CuE caused an accumulation of acidic vesicles, although it did not rescue acridine orange staining in MPP^+^-treated cells (CuE + MPP^+^). This suggests that CuE may act on the autophagy-lysosomal pathway. We then analysed the presence of autophagic vacuoles using Cyto-ID, a green fluorescent dye that accumulates in autophagic vacuoles but not lysosomes [61–63] (Figures 7(a) and 7(b)). Although CuE treatment did not alter the overall number of Cyto-ID-positive vacuoles (Figure 7(a)), it provoked the accumulation of large (>1 *μ*m) vacuoles in neuronal PC12 cells (Figure 7(b)). In addition and in contrast to the staining of acidic vacuoles with acridine orange shown in Figure 6, CuE prompted the accumulation of large Cyto-ID-positive vacuoles (>1 *μ*m) even in the presence of MPP^+^ (CuE + MPP^+^), suggesting that CuE may regulate autophagy upstream of lysosomes. Rapamycin, a known inducer of autophagy, was used as positive control for Cyto-ID staining. Altogether, these results suggest that CuE regulates the autophagolysosomal pathway.

**Figure 8:**
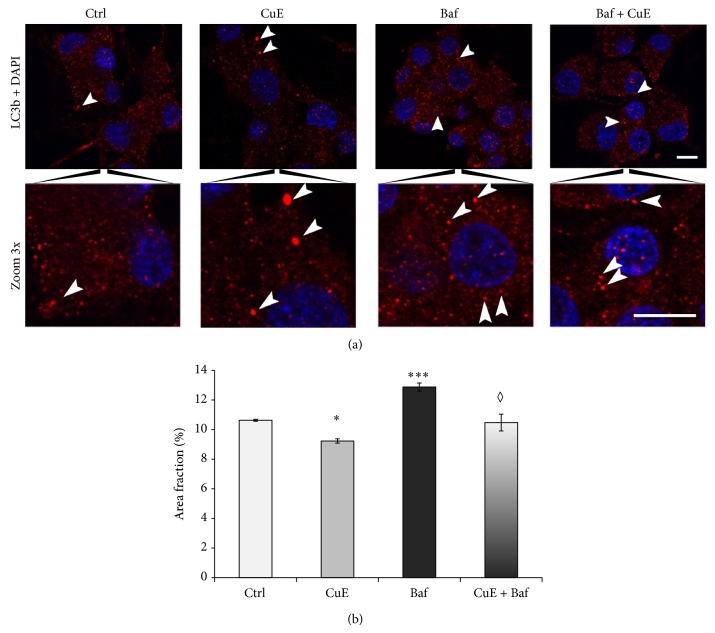
(a) Immunofluorescence detection of LC3b in neuronal PC12 cells. Punctate structures stained for LC3b represent specific autophagic vacuoles. Scale bar = 50 *μ*m. (b) Histogram. CuE treatment did not increase the total number of LC3b-positive autophagosomes but rather slightly decreased their presence. Bafilomycin (Baf), an inducer of autophagy, was used as a positive control. CuE treatment before Baf (Baf + CuE) administration clearly reduced the Baf-induced formation of LC3b-positive autophagosomes. Data are expressed as percentage of cellular surface occupied by LC3b vacuoles (area fraction) and are means ± S.E.M. *n* = 3. ^***^ *P* < 0.001 and ^*^ *P* < 0.05 versus Ctrl. ^◊^ *P* < 0.05 versus Baf.

**Figure 9:**
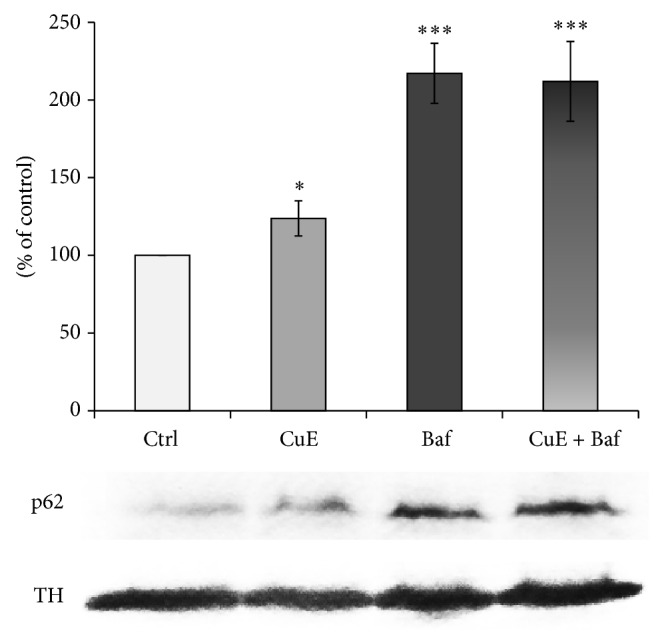
p62 protein expression as revealed by western blot. CuE significantly increases p62 expression, a cargo protein involved in autophagic flux, Baf induces a strong expression of p62, and the administration of CuE + Baf does not additionally increase p62 protein expression. TH was used to normalise p62. Values are the average of 3 samples from 3 independent experiments for a total of 9 measurements. Data are expressed as means ± S.E.M. *n* = 3. ^***^ *P* < 0.001 and ^*^ *P* < 0.05 versus Ctrl.

To directly address the effect of CuE on autophagosome formation, we then tested its effect on the accumulation of vesicles positive for the specific autophagosomal marker LC3 [58, 59]. Upon induction of autophagy, LC3 becomes lipidated, associates with a nascent autophagosomes, and stays associated with the autophagosome until it is degraded within a lysosome. Therefore, we stained neuronal PC12 cells for LC3 and determined the presence of LC3-positive autophagic vacuoles by immunofluorescence (Figures 8(a) and 8(b)). On its own, CuE treatment decreased the number of LC3-positive autophagosomes, suggesting that CuE inhibits autophagy (Figure 8(b)). Importantly, inhibition of lysosomal activity with bafilomycin to prevent degradation of the autophagosomes failed to rescue LC3-positive vesicles in CuE-treated cells. These results suggest that the decrease in autophagosomes observed in CuE-treated cells is the consequence of reduced autophagy rather than an increase in autophagic flux. To further demonstrate that CuE decreases autophagy, we measured the expression of the autophagy substrate p62 by western blotting (Figure 9). Consistent with an inhibitory effect on autophagy, CuE caused the accumulation of p62 in neuronal cells. As with LC3-positive autophagosomes, inhibition of lysosomal acidification with bafilomycin (Baf, Figure 9) increased p62 levels in control cell but did not further increase them in CuE-treated cells. Altogether, our results indicate that CuE decreases autophagic flux.

### 3.4. CuE Reverts Abnormal Relocalization of Lysosomes Induced by MPP^+^ Treatment

**Figure 10:**
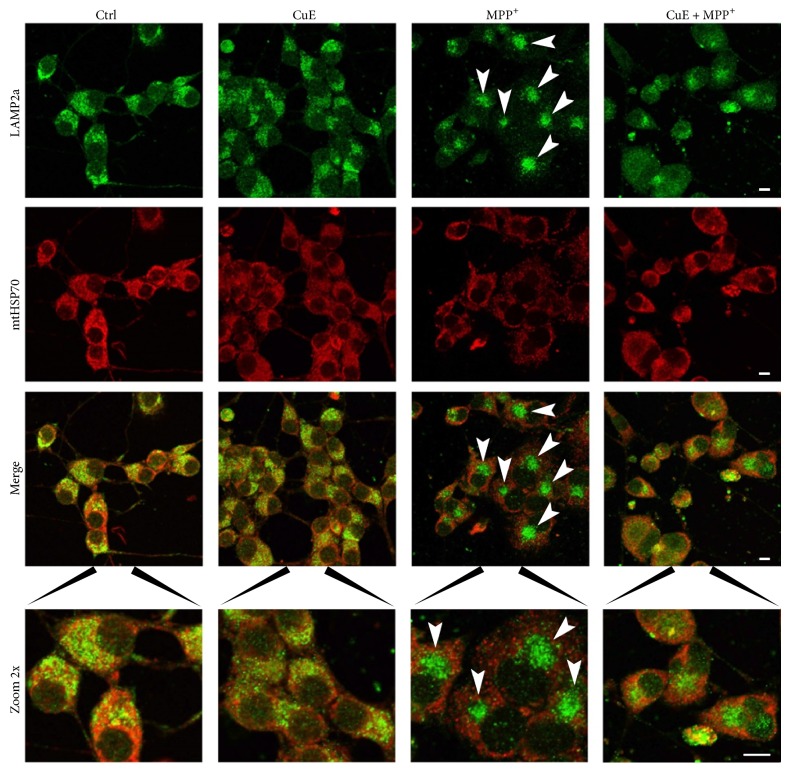
Immunofluorescence pictures illustrating lysosome and mitochondria localization. Neuronal cells were double-stained for LAMP2a, a specific lysosomal marker (green fluorescence), and mtHSP70, a specific mitochondrial marker (red fluorescence). MPP^+^ condition shows delocalisation of lysosomes (green staining and arrowheads) in dense clusters near the nuclei, resulting in a staining pattern dramatically different from the other conditions. Administration of CuE appears to rescue in part normal lysosome localization. Microphotographs are representative of 3 different experiments. Scale bar = 10 *μ*m.

We then tested the effect of CuE on the subcellular distribution of lysosomes and mitochondria. These organelles were visualised using an antibody against the lysosomal membrane protein LAMP2a or against the mitochondrial matrix protein mtHSP70 (Figure 10). MPP^+^ treatment resulted in a strong clustering of lysosomes (green) in the perinuclear region of the cells (Figure 10, MPP^+^, arrowheads). Figure 10 shows mitochondria stained with mtHSP70 in red (second line) and also illustrates that mitochondria are pushed away from the perinuclear region by lysosomal clusters (Figure 10, merge and zoom 2x). While CuE failed to rescue the number of acridine orange acidic vesicles, in MPP^+^-treated cells (Figure 6), it reversed lysosomal clustering as illustrated in Figure 10 (CuE + MPP^+^, zoom 2x) and increased the number of large Cyto-ID-positive vesicles (Figure 7, histogram). These results indicate that while CuE does not directly promote lysosomal acidification, it may regulate the formation of other types of autophagolysosomal vesicles that correlate with improved survival.

### 3.5. CuE Does Not Visibly Alter the Neuronal Cytoskeleton

**Figure 11:**
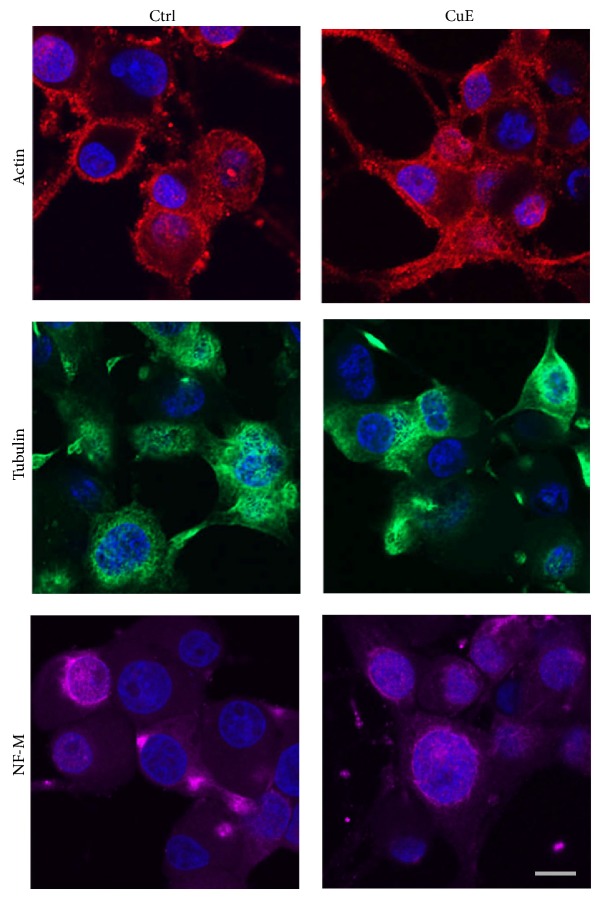
Immunofluorescence detection of several cytoskeletal proteins in CuE- or vehicle-treated neuronal cells (Ctrl). Cells were stained for *β*-actin (red), *β*3-tubulin (green), or neurofilament-M (NF-M) (purple). Hoechst 33342 (blue) was used to counterstain all nuclei. CuE treatment for 24 hours does not visibly alter the neuronal cytoskeleton. Microphotographs are representative of 3 different experiments. Scale bar = 10 *μ*m.

Since autophagy requires the presence of an intact cytoskeleton [67], we analysed the effect of CuE on neuronal PC12 cell cytoskeleton. As shown in Figure 11, CuE did not visibly affect the actin cytoskeleton, suggesting that the accumulation of autophagic vacuole is not the consequence of an altered actin cytoskeleton (Figure 11, red staining). As organelle movement normally depends on microtubules, we also analysed the influence of CuE on *β*3-tubulin staining. As with actin, a 24 h treatment with 10^−10^ M CuE did not visibly alter microtubules (Figure 11, green staining), nor intermediate filaments NF-M (purple staining), indicating that, at the concentration used, CuE does not affect the cellular cytoskeleton in our postmitotic cellular paradigm.

## 4. Discussion

This study details for the first time the neuroprotective effects of CuE, a molecule from the cucurbitacin family. Cucurbitacins, phytosterols found mostly in cucurbits, are currently studied for their important anticancer potential [41, 43, 68, 69]. Some of these compounds also possess interesting anti-inflammatory and antioxidant properties in mitotic cell lines [70, 71]. CuE, in particular, has been proven to impact signal transduction and activation of transcription-3 (STAT3) effect [40] and show an important actin-binding activity that stabilizes microfilaments [42]. This leads to cell cycle arrest and apoptosis in rapidly dividing cells, such as tumor cells [37]. Besides, CuE has been reported to suppress cell invasion and metastasis [68] and does not affect apoptosis in human lymphocytes as compared to prostate adenoma and breast cancer cell lines [70].

Polyphenols and phytosterols have been widely studied for their neuroprotective properties often mediated by their antioxidant activity [29, 55, 72]. A variety of molecules such as resveratrol, quercetin, and sesamin also exert antiapoptotic and anti-inflammatory roles in nonmitotic cells, as demonstrated by others and our previous work [47, 48, 73–75]. Altogether their prosurvival activities on neuronal degeneration might contribute to the developing of new avenues for complementing current therapies for neurodegenerative diseases. To our knowledge, CuE has never been used in a neurodegeneration model of postmitotic neuronal cells and our results are the first to endorse its neuroprotective properties and propose original mechanisms for its activity, such as the modulation of cellular macroautophagy.

We performed our experiment in postmitotic DAergic neurons, NGF-differentiated PC12 cells. This cellular paradigm has been extensively used by us and others to demonstrate that several polyphenols and lignans are indeed neuroprotective by reducing apoptosis and oxidative stress [48, 55, 76–80]. After NGF administration, PC12 cells adopt a neuronal-like phenotype as manifested by secretion of high levels of dopamine and the expression of TH, DAT, neurofilaments, and estrogen receptor-alpha and estrogen receptor-beta [28, 81–83].

Abundant literature reports CuE cytotoxic effects on tumor cells lines leading to cell-arrest [37, 40, 42]. In our postmitotic model, directly administrating CuE at a low dose, (10^−10^ M) to the culture medium of neuronal cells, revealed important neuroprotective properties, as demonstrated by CuE's ability to prevent MPP^+^-induced cell death. This is not the first time that phytochemicals show apparent paradoxical effects [47, 76]. It is reasonable to speculate that low doses of CuE may be involved in activating adaptive responses in postmitotic cellular model. Literature reports prosurvival antioxidant properties of polyphenols and phytosterols. Following this line of evidence, we performed oxidative stress assays in our DAergic cellular model to help pinpoint the mechanism of CuE-mediated neuroprotection. In our cellular paradigm, 10^−10^ M CuE did not possess any significant antioxidant effect, as shown by both DHR oxidization assay and superoxide anion fluorescence (MitoSox assay). A similar intriguing result has been recently reported with cucurbitacin B and cucurbitacin I in a tumor model, linking ROS production with autophagy induction in cucurbitacin-treated cells [84].

As autophagy impairment is observed in several syndromes of neurodegeneration such as PD [9, 85, 86], the autophagy-lysosome pathway has been proposed as a target for neurodegeneration therapies [11, 20, 87]. On the other hand, autophagy activation has been shown to be detrimental in other models of neuronal loss such as following a stroke [88, 89]. Therefore, while autophagy activation can promote survival when the accumulation of damaged cellular components is the primary issue, it can also have detrimental consequences for neurons.

In this context, it is noteworthy that the neuroprotective effects of CuE in a neuronal model of MPP^+^-induced cell death are associated with a decrease in autophagy. In fact, both the decrease in LC3-positive autophagosomes and the accumulation of p62 are consistent with a decrease in autophagy initiation and possibly impaired lysosomal degradation of autophagosomes. This is suggested by the observation that bafilomycin failed to recover LC3 vesicles or p62 protein expression. On the other hand, CuE increased the presence of large autophagic vacuoles (>1 *μ*m) and rescued lysosomal distribution in MPP^+^-treated cells, suggesting that the decrease in lysosomal delivery of autophagosomes caused by CuE could rescue some aspects of lysosomal function. As lysosomes play an important role in neurons [90], this could explain, at least in part, the neuroprotective role of CuE.

In dynamic mitotic cell lines, such as tumor cells, CuE has been reported to promote the remodeling of actin filaments leading to the formation of actin clusters [42, 44]. However, we did not detect any changes in the appearance of the actin cytoskeleton in our paradigm following CuE administration. It is however difficult to conclude that CuE does not alter the cellular cytoskeleton since other studies reporting CuE-induced cytoskeletal changes have used higher doses of CuE in different cellular system [42, 44]. Thus, we cannot exclude the possibility that CuE affects the cytoskeleton in a more subtle manner in our system. In addition, MPP^+^ may impair microtubules dynamics, as recently reported in [91, 92]. Our results do not exclude the possibility that the neuroprotective effects of CuE may be related to a stabilizing action exerted on the neuronal cytoskeleton.

In conclusion, autophagy regulation as a means of neuroprotection is rather promising, though it is important to consider its “ying-yang” role. Indeed, as much as the recycling of toxic organelles and proteins might benefit cell, too much autophagy may also result in autophagic cell death [93, 94]. Therefore, the use of molecules targeting the autophagic pathways will require important fine-tuning of dosages. Our data revealing a modulation of the autophagy pathways when using low doses of CuE and a particular role on lysosomal function propose that this terpenoid molecule could have powerful roles in the cellular dynamic underlying neurodegeneration and neuroprotection.
